# Functional Characterization and Genotype‐Phenotype Correlation of Androgen Receptor Variants in Patients With Androgen Insensitivity Syndrome

**DOI:** 10.1155/humu/1882678

**Published:** 2026-10-05

**Authors:** Di Mao, Lijun Fan, Yueni Zhang, Chunxiu Gong

**Affiliations:** ^1^ Department of Pediatrics The Sixth Affiliated Hospital of Harbin Medical University Harbin Medical University Harbin China hrbmu.edu.cn; ^2^ Department of Endocrinology Genetics Metabolism Beijing Children′s Hospital Capital Medical University National Center for Children′s Health Beijing China ccmu.edu.cn; ^3^ Department of Medical Genetics College of Basic Medical Sciences Harbin Medical University Harbin China hrbmu.edu.cn

**Keywords:** androgen insensitivity syndrome, *AR* variants, functional assays, pathogenicity

## Abstract

Androgen insensitivity syndrome (AIS) is a 46,XY disorder of sex development caused by pathogenic variants in the androgen receptor (*AR*) gene. This study is aimed at characterizing the clinical features and investigating the functional consequences of *AR* variants identified in a Chinese AIS cohort. A systematic literature review was conducted to compare existing functional assays and pathogenic mechanisms. Multiple in silico tools were applied to predict the functional impact of *AR* variants, followed by in vitro functional analyses of missense variants in MLTC‐1 cells. Protein expression was evaluated by Western blotting, subcellular localization by immunofluorescence, and transcriptional activity using a pGL4.11‐(ARE)_3_‐TATA‐luc dual‐luciferase reporter assay. Among reported *AR* missense variants, only 28% (141/504) had undergone functional evaluation, comprising 274 assays, mainly ligand‐binding and transcriptional activity analyses. In the present cohort, 33 patients with AIS harboring *AR* variants were analyzed, including 15 patients with complete AIS and 18 patients with partial AIS. Seventeen missense variants were further evaluated using in vitro functional assays. Reduced AR protein expression was observed in 82% (14/17) of variants, impaired nuclear localization in 59% (10/17), and reduced transcriptional activity in 53% (9/17), whereas increased transcriptional activity was observed in 12% (2/17) of variants. Nuclear localization was positively correlated with transcriptional activity, and residual transcriptional activity was positively associated with EMS scores. These findings demonstrate that *AR* variants cause AIS through heterogeneous molecular mechanisms and that residual AR function contributes to phenotypic variability.

## 1. Introduction

Androgen insensitivity syndrome (AIS) is an X‐linked disorder caused by variants in the androgen receptor (*AR*) gene that impair AR function and was first described by Morris in 1953 [1]. The estimated incidence ranges from 1 in 40,800 to 1 in 9000. The *AR* gene encodes a ligand‐dependent transcription factor consisting of eight exons and 920 amino acids. The protein comprises four functional regions: the N‐terminal domain (NTD), DNA‐binding domain (DBD), hinge region, and ligand‐binding domain (LBD) [2]. In the absence of ligand binding, AR resides in the cytoplasm as part of a multiprotein chaperone complex. Upon binding to endogenous androgens, such as testosterone or dihydrotestosterone (DHT), or to synthetic androgens, AR translocates into the nucleus as a ligand‐activated transcription factor. It then binds to androgen response elements (AREs) in target gene promoters to regulate androgen‐mediated biological processes [3].

Variants impair AR structure or function, rendering target tissues insensitive to androgens critical for virilization [4, 5]. Based on residual AR activity, AIS is clinically classified into complete (CAIS), partial (PAIS), and mild (MAIS) forms. CAIS presents with a typical female phenotype. PAIS exhibits variable degrees of under‐virilization, including micropenis, hypospadias, and cryptorchidism at birth. MAIS represents the mildest form, characterized by normal male external genitalia but impaired androgen action, manifesting as reduced body hair, gynecomastia, or a small penis [6].

To date, more than 800 *AR* variants associated with AIS have been identified. Missense variants are the most common and predominantly occur within the LBD. Although commonly predicted pathogenic by in‐silico tools, these algorithms lack specificity and over‐call damaging effects; therefore, computational data alone are insufficient for clinical diagnosis [7]. In this study, we systematically analyzed the clinical phenotypes and molecular genetic characteristics of 33 individuals with AIS harboring different *AR* variants and performed in vitro functional characterization of 17 missense variants. By integrating variant type and domain distribution with AR protein expression, nuclear localization, transcriptional activity, and clinical phenotypic severity, we comprehensively evaluated genotype‐function‐phenotype relationships in AIS. We aimed to elucidate the relationship between residual AR function and phenotypic heterogeneity and to provide functional evidence for the pathogenic interpretation of *AR* variants and clinical assessment of AIS.

## 2. Methods

### 2.1. Literature Review

We searched the Human Gene Mutation Database (HGMD; updated to April 2024) to identify published studies that performed functional analyses to evaluate the pathogenicity of missense *AR* variants. These studies included androgen‐binding assays, transcriptional activation assays, and additional functional investigations such as protein expression and nuclear translocation analyses. Detailed information is provided in Table S1.

### 2.2. Case Report and Genetic Analysis

The 33 patients with AIS included in this study were identified from an institutional longitudinal AIS database. The clinical and genetic characteristics of some patients have been previously described in our cohort study [8]. In the present study, we performed an integrated analysis of clinical phenotypes and genetic characteristics and further conducted in vitro functional characterization of 17 missense *AR* variants.

AIS was classified as CAIS or PAIS based on the degree of external genital masculinization at the first clinical evaluation. The external genital phenotype was assessed using the external masculinization score (EMS), which incorporates penile length, urethral meatus position, labioscrotal fusion, and gonadal location. EMS was calculated according to previously established criteria and used to quantify the degree of virilization [9]. Penile length was evaluated using age‐specific reference values, and micropenis was defined as a stretched penile length below −2.5 standard deviations (SDs) for age‐matched healthy Chinese children [10].

Variant pathogenicity was classified according to the standards and guidelines of the American College of Medical Genetics and Genomics (ACMG), using the Franklin Genoox platform (https://franklin.genoox.com/clinical-db/home) with manual expert review. For missense variants, pathogenicity assessment was based on an integrated scoring framework incorporating 23 in silico prediction tools and databases. A detailed list of the computational tools is provided in Table S2.

### 2.3. In Vitro Functional Assays

#### 2.3.1. Plasmid Construction

Wild‐type AR expression plasmids (pCAG‐EGFP‐*AR*‐WT) and a dual‐luciferase reporter plasmid (pGL4.11‐(ARE)₃‐TATA‐Luc) were constructed. Using pCAG‐EGFP‐*AR*‐WT as a template, site‐directed mutagenesis was performed to generate 17 *AR* variant expression constructs: p.Y269S, p.G578E, p.P672L, p.D691E, p.Q734K, p.M746K, p.Y782N, p.E804V, p.L806M, p.L811V, p.G821E, p.N834Y, p.Y835H, p.L860F, p.S885L, p.V888A, and p.I907L. To generate the androgen‐responsive reporter plasmid, three tandem repeats of an ARE (ARE; 5 ^′^‐AGAACAGCAAGTGTTCT‐3 ^′^) were inserted into the pGL4.11 reporter vector, resulting in pGL4.11‐(ARE)₃‐TATA‐Luc, as previously described [11]. Primer sequences are listed in Table S3.

### 2.4. Dual‐Luciferase Reporter Assay

MLTC‐1 cells were obtained from the National Collection of Authenticated Cell Cultures (NCACC), Chinese Academy of Sciences (Shanghai, China), and cultured in RPMI 1640 medium supplemented with 20% fetal bovine serum at 37°C in a humidified atmosphere containing 5% CO₂. Cells were routinely tested and confirmed to be free of mycoplasma contamination. MLTC‐1 cells were seeded in 12‐well plates and cotransfected using Lipofectamine 3000 (Thermo Fisher Scientific, L3000015) with wild‐type or *AR* variant expression plasmids (200 ng/well), the firefly luciferase reporter plasmid containing AREs (200 ng/well), and the Renilla luciferase plasmid pGL4.74 (30 ng/well) as an internal control. Forty‐eight hours after transfection, luciferase activities were measured using the Dual‐Luciferase Reporter Assay System (Promega, E2920) according to the manufacturer′s instructions. Firefly luciferase activity was normalized to Renilla luciferase activity and expressed as relative luciferase activity. Each sample was analyzed in triplicate, and all experiments were independently repeated at least three times.

### 2.5. Western Blotting

MLTC‐1 cells were transfected with pCAG‐EGFP‐*AR* expression plasmids encoding wild‐type or AR variant proteins. Whole‐cell lysates were prepared using 1% SDS lysis buffer. Proteins were separated by Bis‐Tris gel electrophoresis and transferred to PVDF membranes.

Membranes were incubated overnight at 4°C with primary antibodies against AR (Cell Signaling Technology, D6F11) and GFP (ABclonal, AE011), followed by washing with TBST three times (5 min each). Membranes were then incubated with HRP‐conjugated secondary antibodies (Proteintech Group, RGAR001) for 1 h at room temperature and washed again with TBST. Protein bands were visualized using enhanced chemiluminescence substrate (Tanon, 180‐5001) and detected with a BIO‐RAD imaging system. Band intensities were quantified using ImageJ software. All experiments were independently repeated three times.

### 2.6. Immunofluorescence

MLTC‐1 cells were transfected with pCAG‐EGFP‐*AR* expression plasmids encoding wild‐type or AR variant proteins (pIRES2‐EGFP‐*AR*‐WT or pIRES2‐EGFP‐*AR*‐MUT). After 48 h, cells were fixed with 4% paraformaldehyde for 15 min and blocked with 0.5% Triton X‐100‐containing blocking buffer for 2 h. Cells were incubated overnight with a primary anti‐AR antibody (Cell Signaling Technology, D6F11), followed by incubation with a fluorescent secondary antibody (Invitrogen, A21428) for 2 h. Nuclei were counterstained with DAPI (Dojindo Laboratories, D523) for 10 min. Images were acquired using a confocal laser‐scanning microscope (Zeiss) with a 40× objective. For each variant, 3–6 images were captured.

### 2.7. Statistical Analysis

Functional assay data, including protein expression, nuclear localization, and transcriptional activity, are presented as mean ± SD. Relative transcriptional activity was calculated as the percentage of wild‐type activity. Differences between each AR variant and the wild‐type control were analyzed using one‐way analysis of variance (ANOVA). Statistical analyses were performed using GraphPad Prism Version 10.1.2.

Correlations between functional parameters and clinical phenotypes were assessed using Spearman′s rank correlation analysis. Linear regression analysis was performed to evaluate the relationship between nuclear localization and transcriptional activity. Associations between categorical variables, including AR functional domains or variant classes and clinical phenotypes (CAIS/PAIS classification and sex assignment), were evaluated using Fisher′s exact test. A two‐sided *p* value < 0.05 was considered statistically significant.

## 3. Results

### 3.1. Literature Review

To summarize current evidence on the pathogenicity of *AR* missense variants, we performed a systematic literature review. In total, 504 missense variants were identified, most of which were located in the LBD (325/504), followed by the NTD (87/504), DBD (82/504), and the hinge region (10/504) (Figure S1A). AIS patients accounted for the majority of cases (366/504), including 160 with CAIS, 113 with PAIS, and 11 with MAIS; the remaining patients were not clearly classified (Figure S1B).

A total of 274 functional assays were performed for 141 distinct *AR* missense variants (Table S1). Androgen‐binding assays were used in 121/274 experiments. The most commonly used cell model was patient‐derived fibroblasts (64/121), followed by COS cells (32/121), CHO cells (8/121), and Hep3B cells (7/121). These assays involved variants identified in 62 patients with CAIS, 5 patients with MAIS, 39 patients with PAIS, and 3 patients with spermatogenic disorders. Among the 62 patients with CAIS, 11% (7/63) showed normal androgen‐binding capacity (normal *K*_*d*_, *B*_max_, and dissociation), whereas 54% (34/63) showed complete loss of binding. Among the 5 patients with MAIS, 1 showed normal binding, 1 showed complete loss of binding, and the remaining three exhibited varying degrees of reduced binding capacity. In patients with PAIS, 26% (10/39) had normal binding, whereas 15% (6/39) showed complete loss of binding. Among infertile patients, 67% (2/3) retained normal androgen‐binding capacity.

Transcriptional activity assays were performed in 92 experiments, most commonly using the MMTV reporter system (40/92), followed by ARE/GRE‐based systems (36/92). Variants from 47 CAIS cases, 40 PAIS cases, 1 MAIS case, and 4 infertile patients were evaluated. In CAIS, 26% (12/47) of variants showed undetectable transcriptional activity, 6% (3/47) retained normal activity, and 68% (32/47) retained 3%–80% of WT activity. Among these, 16% (5/32) showed partial rescue of transcriptional activity upon exposure to high concentrations of testosterone or DHT. In PAIS, 7.5% (3/40) of variants showed undetectable transcriptional activity, 7.5% (3/40) retained normal activity, and the remaining variants retained 10%–80% of WT activity. The single MAIS‐associated variant retained approximately 60% of WT activity, which could be rescued by high doses of DHT. Among infertile patients, 25% (1/4) retained normal transcriptional activity, whereas the others retained 70%–80% of WT activity.

In addition to androgen‐binding and transcriptional activity assays, several other in vitro functional studies were reported. Western blot analysis was used to assess protein expression for *AR* variants identified in 27 patients with CAIS, showing that most variants (18/27) had protein expression levels comparable to WT. Immunofluorescence analyses were performed for 14 *AR* variants to evaluate nuclear translocation. Most variants did not affect subcellular localization (71% [5/7] in CAIS; 86% [6/7] in PAIS) (Figure S1C; Table S1).

### 3.2. Study Cohort

The diagnostic classification and distribution of *AR* variants in the AIS cohort are summarized in Table 1. The cohort comprised 33 patients with a confirmed 46,XY karyotype, including 15 with CAIS and 18 with PAIS, with a median age at diagnosis of 1.9 years (range: 0.3–16.2 years, Table S4). Among CAIS patients, 75% (10/15) harbored nonsense or frameshift variants leading to premature truncation within the NTD, LBD or hinge region, whereas 25% (5/15) carried missense variants, all located in the LBD. Among PAIS patients, 72% (13/18) carried missense variants, of which 85% (11/13) were located in the LBD. Additionally, 17% (3/18) of PAIS patients carried nonsense or frameshift variants truncating the NTD or LBD, and two PAIS patients harbored an in‐frame indel variant (Figure S2).

**Table 1:** Characterization of the AIS patients harboring *AR* variants.

Patient	Age at presentation	Classification	External genitalia	Gonadal findings	Sex assignment/reassignment	Genetic characterization		
						Variant	ACMG classification (criteria)	Parental inheritance
1	8m26d	PAIS	Penile length: 2.3 × 0.8 cm; urethral meatus located on the penile shaft	Bilateral scrotal testes (1 mL each)	M	p. W399X	P (PVS1 + PM2_supporting+PS2)	De novo
2	4 m	PAIS	Penile length: 0.5 × 0.5 cm; urethral meatus located on the scrotum; bifid scrotum	Bilateral inguinal testes	F → M	p. Y552X	P (PVS1 + PM2_supporting+PS2)	De novo
3	4y3m	PAIS	Penile length: 2.0 × 1.5 cm; urethral meatus located on the penile shaft	Bilateral scrotal testes (2 mL each)	M	p. G578E	LP(PM2_supporting+PP2 + PM5 + PP3 + PS3_supporting)	F: WT; M: het
4	7y	CAIS	Female external genital appearance	Bilateral inguinal testes	F	p. T653Lfs ^∗^8	LP (PVS1 + PM2_supporting)	F: WT; M: het
5	1y3m	CAIS	Female external genital appearance	Bilateral inguinal testes	F	p. V662Lfs ^∗^11	LP (PVS1 + PM2_supporting)	F: WT; M: het
6^a^	1y7m	PAIS	Penile length: 0.8 × 0.8 cm; urethral meatus located in the perineum	Bilateral scrotal testes (1 mL each)	M	p. P672L	LP (PM1 + PP2 + PM2_supporting_supporting+PM5)	F: WT; M: het
7	5y6m	CAIS	Female external genital appearance	Bilateral inguinal testes	F	p. Q69X	LP (PVS1 + PM2_supporting)	F: WT; M: het
8	1y11 m	PAIS	Clitoris‐like phallus with penoscrotal transposition	Bilateral scrotal testes (1–2 mL each)	M	p. D691E	LP(PS1 + PM1 + PP2 + PM2_supporting+PS3_supporting)	F: WT; M: het
9	1y1m	CAIS	Female external genital appearance	Bilateral inguinal testes	F	p. Q694X	LP (PVS1 + PM2_supporting)	F: WT; M: het
10	1y9m	CAIS	Female external genital appearance	Bilateral inguinal testes	F	p. R711Tfs ^∗^57	P (PVS1 + PM2_supporting+PS2)	De novo
11	1y2m	CAIS	Female external genital appearance	Bilateral inguinal testes	F	p. Q712X	P (PVS1 + PM2_supporting+PS2)	De novo
12	2y	CAIS	Female external genital appearance	Bilateral inguinal testes	F	p. Q734K	LP (PM1 + PP2 + PM2_supporting+PM5 + PP3 + PS3_ supporting)	F: WT; M: het
13	11 m	PAIS	Penile length: 2.0 × 1.0 cm; urethral meatus located on the scrotum	Bilateral scrotal testes (2 mL each)	M	p. Q734K	LP (PM1 + PP2 + PM2_supporting+PM5 + PP3 + PS3_ supporting)	F: WT; M: het
14	16y2m	CAIS	Female external genital appearance	Testis located in the iliac fossa	F	p. M746K	P(PM1 + PP2 + PM2_supporting+PM5 + PP3 + PS3_moderate)	F: WT; M: het
15	12y	PAIS	Postoperative status after orchiopexy and hypospadias repair	Postoperative status after orchiopexy and hypospadias repair	M	p. Y782N	LP (PM1 + PP2 + PM2_supporting+PM5 + PP3 + PS3_ supporting)	F: WT; M: het
16	4 m	CAIS	Female external genital appearance	Bilateral inguinal testes	F	p. E804V	P (PM1 + PP2 + PM2_supporting+PM5 + PP3 + PS3_ moderate)	F: WT; M: het
17	7m20d	PAIS	Penile length: 1.7 × 1.2 cm; urethral meatus located on the penile shaft	Bilateral scrotal testes (2 mL each)	M	p. L806M	P (PM1 + PP2 + PM2_supporting+PS2 + PP3 + PS3_ supporting)	De novo
18	4 m	PAIS	Penile length: 1.0 × 1.0 cm; urethral meatus located at the penile base	Bilateral scrotal testes (1–2 mL each)	M	p. L811V	LP (PM1 + PP2 + PM2_supporting+PM5 + PP3 + PS3_ supporting)	F: WT; M: het
19	6y	PAIS	Penile length: 3.1 × 1.2 cm; bifid scrotum; urethral meatus located at the penile base	Bilateral inguinal testes	F	p. G821E	LP (PP3 + PM2_supporting+PM5 + PP2 + PS3_ moderate)	ND
20	7y	CAIS	Female external genital appearance	Left testis located in the inguinal canal; right testis located in the iliac fossa	F	p. F827Ifs ^∗^3	LP (PVS1 + PM2_supporting)	F: WT; M: het
21	4y9m	PAIS	Penile length: 2.0 × 0.8 cm; urethral meatus located at the penile base; bifid scrotum	Bilateral scrotal testes (2 mL each)	M	p. 829_830delDEinsE	LP (PM4 + PM5 + PM2_supporting+PM1)	F: WT; M: het
22	1y	PAIS	Penile length: 2.0 × 1 cm	Bilateral scrotal testes (2 mL each)	M	p. N834Y	LP (PM1 + PP2 + PM2_supporting+PP3 + PS3_ moderate)	F: WT; M: het
23	1y11 m	CAIS	Female external genital appearance	Testis located in the iliac fossa	F	p. Y835H	P (PM1 + PP2 + PM2_supporting+PM5 + PS3_ moderate)	F: WT; M: het
24	1y9m	PAIS	Penile length: 1.8 × 0.8 cm; bifid scrotum; urogenital sinus opening located in the perineum	Right testis located in the scrotum (2 mL); left testis located in the inguinal canal	F	p. L860F	LP (PM1 + PP2 + PM2_supporting+PP3 + PS2 + PS3_ supporting)	De novo
25	1y11 m	CAIS	Female external genital appearance	Bilateral inguinal testes	F	p. Q868X	LP (PVS1 + PM2_supporting)	F: WT; M: het
26	13y7m	PAIS	Penile length: approximately 3.5 cm; urethral and vaginal openings were present	Bilateral inguinal testes	F	p. 881_882delLLinsL	LP (PM4 + PM5 + PM2_supporting+PM1)	F: WT; M: het
27	2y5 m	PAIS	Penile length: 0.8 cm; urogenital sinus opening	Bilateral inguinal testes	M	p. H886Nfs ^∗^4	LP (PVS1 + PM2_supporting)	ND
28	2y	CAIS	Female external genital appearance	Left testis located at the internal inguinal ring; right testis located in the iliac fossa	F	p. S885L	LP (PM1 + PP2 + PM2_supporting+PP3 + PS3_ moderate)	F: WT; M: het
29	3y6m	PAIS	Penile length: 2.0 × 1.0 cm; urethral meatus located at the subcoronal region	Bilateral scrotal testes (2 mL each)	M	p. V888A	VUS (PM1 + PP2 + PM2_supporting+PP3)	F: WT; M: het
30	3y8m	PAIS	Penile length: 2.5 × 1.2 cm; urethral meatus located at the penile base	Bilateral scrotal testes (1–2 mL each)	M	p. I907L	LP (PM1 + PP2 + PM2_supporting+PS2 + PP3 + PS3_ supporting)	De novo
31	5 m	CAIS	Female external genital appearance	Bilateral inguinal testes	F	p. Y225X	LP (PVS1 + PM2_supporting)	F: WT; M: het
32	5 m	CAIS	Female external genital appearance	Bilateral inguinal testes	F	p. Y225X	LP (PVS1 + PM2_supporting)	F: WT; M: het
33	11y	PAIS	Penile length: 2.6 × 1.2 cm; urethral meatus located at the penile base	Bilateral scrotal testes (2 mL each)	M	p. Y269S	VUS (PM2_supporting+PP2 + PP3 + PS3_ supporting)	ND

*Note:* Sequence information is based on the following reference sequences or transcripts: AR: NM_000044; MAMLD1: NM_001177465. ACMG criteria for classification of variants pathogenicity: PVS1, very strong evidence of pathogenicity; PS1/2, strong evidence of pathogenicity; PM1–6, moderate evidence of pathogenicity; PP1–5, supporting evidence of pathogenicity.

Abbreviations: F, father; het, heterozygous; LP, likely pathogenic; M, mother; m, month; ND, not determined; P, pathogenic; VUS, variant of unknown significance; y, year.

^a^The patient additionally harbors a maternally inherited *MAMLD1* variant (c.2618C>A, p.A873D), classified as a VUS.

Family segregation analysis was available for 30 patients, revealing that 77% (23/30) of variants were maternally inherited, whereas 23% (7/30) arose de novo. Patient 6 also carried a maternally inherited *MAMLD1* variant (c.2618C>A, p.A873D), classified as a variant of uncertain significance (VUS). Notably, the same *AR* variant (c.2200C>A, p.Q734K) was identified in two unrelated individuals (Patient 12 and Patient 13), who were diagnosed with CAIS and PAIS, respectively.

### 3.3. Association Between *AR* Variant Characteristics and Clinical Phenotypes

We first evaluated whether the location of *AR* variants within different functional regions (LBD vs. non‐LBD regions) was associated with distinct clinical phenotypes. No significant differences were observed between LBD and non‐LBD variants in assigned sex, CAIS/PAIS classification, or external genital characteristics (Table S5).

We further investigated whether *AR* variant class was associated with clinical manifestations by categorizing variants into truncating and nontruncating groups (Figure 1A). Clinical characteristics, including EMS, sex assignment, and CAIS/PAIS classification, were compared between the two groups. No significant differences were observed in EMS scores or sex assignment between truncating and nontruncating variants (Figure 1B,C). However, truncating variants were significantly enriched among patients with CAIS, whereas nontruncating variants were more frequently associated with PAIS (Fisher′s exact test, *p* = 0.005) (Figure 1D).

**Figure 1:**
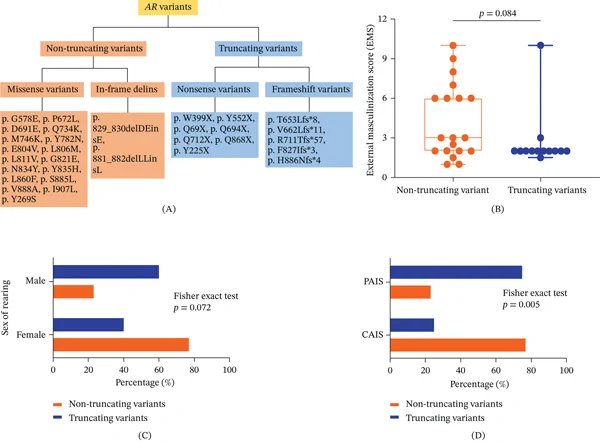
Clinical phenotype analysis according to truncating and nontruncating *AR* variants. (A) Classification of *AR* variants according to variant type. Variants were categorized into truncating variants (nonsense and frameshift variants) and nontruncating variants (missense variants and in‐frame deletions). (B) Comparison of external masculinization score (EMS) between patients carrying truncating and nontruncating *AR* variants. No significant difference was observed between the two groups (Mann–Whitney *U* test, *p* = 0.084). (C) Comparison of sex assignment between patients with truncating and nontruncating *AR* variants. No significant difference was observed (Fisher′s exact test, *p* = 0.072). (D) Comparison of CAIS/PAIS classification between patients with truncating and non‐truncating *AR* variants. Truncating variants were significantly enriched among patients with CAIS, whereas non‐truncating variants were more frequently observed in patients with PAIS (Fisher′s exact test, *p* = 0.005).

### 3.4. In Silico Pathogenicity Prediction

All 17 *AR* missense variants identified in this cohort affected highly evolutionarily conserved residues (Figure S3A). Most missense variants were predicted to be potentially deleterious by more than 18 of the 23 in silico prediction algorithms applied, with the exception of p.Y269S, p.P672L, p.L811V, p.S885L, p.V888A, and p.I907L. Among carriers of these variants, p.S885L was associated with a CAIS phenotype, whereas the others presented with PAIS. The ACMG classification for p.Y269S and p.V888A was VUS (Figure S3B).

### 3.5. Protein Expression

To further assess the pathogenicity of these variants, AR protein expression was examined in MLTC‐1 cells. A protein band corresponding to approximately 110 kDa was detected for most variants, whereas the p.G821E variant exhibited an aberrant band at approximately 80 kDa. Densitometric analysis revealed no significant change in protein expression for p.P672L, p.L806M, or p.V888A. In contrast, protein expression was reduced by approximately 40% for p.Y269S, 35% for p.G578E, 30% for p.D691E, 25% for p.Q734K, 30% for p.M746K, 20% for p.Y782N and p.E804V, 35% for p.L811V and p.G821E, 40% for p.N834Y, 35% for p.Y835H, 75% for p.L860F, 25% for p.S885L, and 40% for p.I907L (Figure 2).

**Figure 2:**
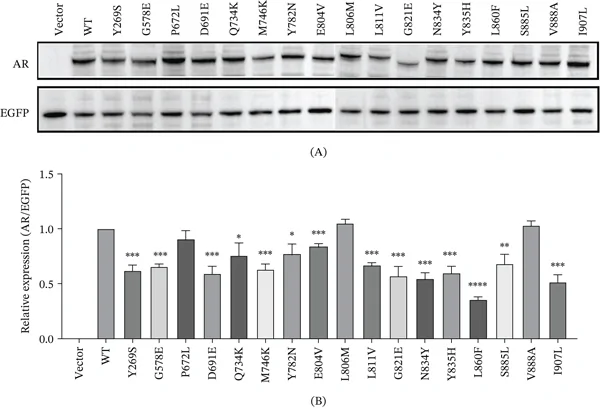
(A) AR protein levels in MLTC‐1 cells transfected with wild‐type or AR variant constructs. (B) Quantification of AR protein expression (mean ± SEM, *n* = 3). EGFP was used as the loading control. *p* values were determined using a one‐way analysis of variance (ANOVA), and *AR* variants were compared with WT. The asterisks indicate statistical significance as follows: *p < 0.05, **p < 0.01, ***p < 0.001, and ****p < 0.0001.

### 3.6. Subcellular Localization

To determine whether *AR* variants affected nuclear translocation, subcellular localization was assessed in MLTC‐1 cells. To evaluate whether *AR* variants affect intracellular localization, we quantified nuclear localization of AR variant proteins by immunofluorescence analysis. Wild‐type AR showed predominant nuclear localization, with approximately 90% of AR signal detected in the nucleus. Similar nuclear localization patterns were observed for p.Y269S, p.G578E, p.P672L, p.D691E, p.Q734K, p.V888A, and p.I907L variants, which showed nuclear localization comparable to wild‐type AR.

In contrast, several variants exhibited impaired nuclear localization. Nuclear localization was reduced to approximately 30% of wild‐type levels for p.M746K and p.G821E, whereas p.E804V retained approximately 50% nuclear localization. Intermediate impairment was observed for p.Y782N, p.N834Y, p.Y835H, p.L860F, and p.S885L, with approximately 60% of AR variant proteins localized in the nucleus. p.L806M and p.L811V retained relatively preserved nuclear localization, with approximately 75% of AR signal detected in the nucleus (Figure 3) and Figure S4).

**Figure 3:**
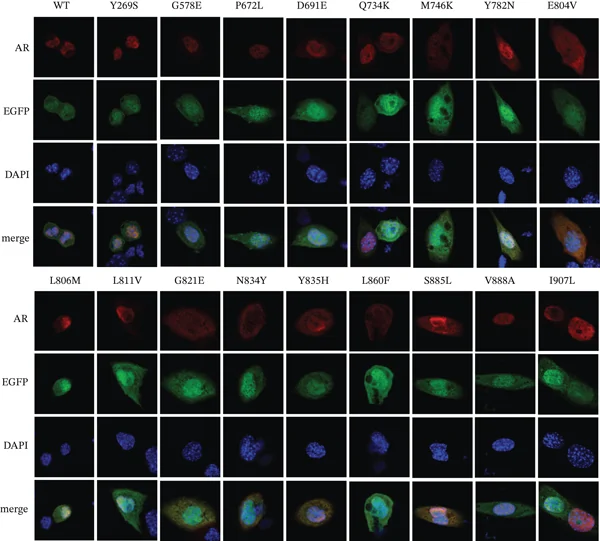
Subcellular localization of wild‐type and AR variant proteins in MLTC‐1 cells. Immunofluorescence staining shows AR (red), EGFP (green), and nuclei (blue, DAPI), together with merged images.

### 3.7. Transcriptional Activity

Finally, we evaluated the transcriptional activity of AR variant proteins using an ARE reporter system, given the broad spectrum of *AR* downstream targets. No significant changes in transcriptional activity were observed for p.Y269S, p.P672L, p.D691E, p.Y782N, p.L811V, or p.L860F. In contrast, transcriptional activity was reduced to approximately 20% of WT for p.M746K, p.E804V, p.G821E, and p.Y835H, and to approximately 50% of WT for p.L806M, p.N834Y, p.S885L, and p.I907L. The p.Q734K variant retained approximately 80% of WT activity. Notably, transcriptional activity was increased for p.G578E and p.V888A, showing approximately 30% and 55% higher activity than WT, respectively (Figure 4).

**Figure 4:**
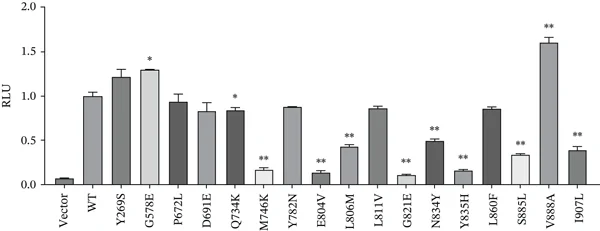
Analysis of the transactivation activity of the *AR* variants by cotransfection assays in MLTC‐1 cells. MLTC‐1 cells were cotransfected with a reporter gene, (ARE)_3_‐ TATA, and an expression vector encoding either an AR variant or wild‐type AR. *p* values were determined using a one‐way analysis of variance (ANOVA), and variants were compared with WT. The asterisks indicate statistical significance as follows: *p < 0.05, **p < 0.01, ***p < 0.001, and ****p < 0.0001.

### 3.8. Relationship Among AR Protein Expression, Nuclear Localization, and Transcriptional Activity

To investigate whether different aspects of AR dysfunction were interconnected, we performed correlation analyses among protein expression, nuclear localization, and transcriptional activity of the 17 *AR* variants. No significant correlation was observed between AR protein expression and nuclear localization (Spearman′s *r* = 0.020, *p* = 0.944) or between protein expression and transcriptional activity (Spearman′s *r* = 0.261, *p* = 0.311). In contrast, nuclear localization showed a significant positive correlation with transcriptional activity (Spearman′s *r* = 0.601, *p* = 0.011) (Figure S5).

### 3.9. Relationship Between Transcriptional Activity and Clinical Phenotype

To determine whether residual AR transcriptional activity was associated with clinical severity, we analyzed the correlation between transcriptional activity and EMS scores. Residual transcriptional activity showed a significant positive correlation with EMS (Spearman′s *r* = 0.573, *p* = 0.016).

Furthermore, variants were categorized according to their residual transcriptional activity into reduced activity (0%–30% of wild‐type activity), preserved activity (30%–100% of wild‐type activity), and increased activity (> 100% of wild‐type activity). The reduced activity group showed significantly lower EMS scores compared with the preserved activity group (*p* = 0.012). No significant difference was observed between the preserved activity group and the increased activity group (*p* = 0.161) (Figure 5).

**Figure 5:**
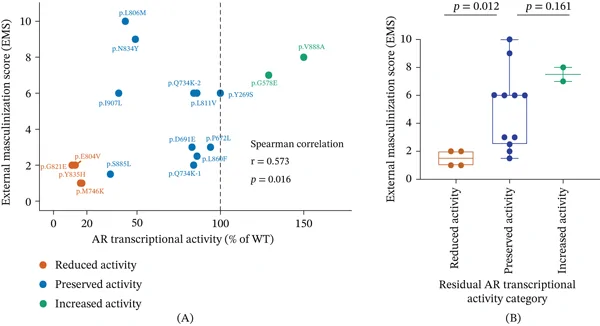
Relationship between AR transcriptional activity and clinical masculinization phenotype. (A) Correlation between AR transcriptional activity and EMS score. Scatter plot showing the relationship between AR transcriptional activity (% of wild‐type activity) and external masculinization score (EMS) among patients carrying functionally characterized *AR* variants. Each dot represents an individual patient. Variants with markedly reduced transcriptional activity tended to be associated with lower EMS scores, whereas variants retaining or showing increased activity were generally associated with milder phenotypes. (B) EMS score according to AR transcriptional activity categories. Distribution of external masculinization scores according to residual AR transcriptional activity categories. Variants were classified into reduced activity (0%–30% of wild‐type activity), preserved activity (30%–100% of wild‐type activity), and increased activity (> 100% of wild‐type activity) groups based on in vitro transcriptional activity assays. Differences in EMS scores between groups were assessed using the Mann–Whitney *U* test.

## 4. Discussion

In this study, we performed an integrated clinical and functional analysis of *AR* variants in patients with AIS. By combining clinical phenotyping, genetic analysis, and in vitro functional assays, we investigated the relationship between *AR* variant‐associated molecular defects and clinical variability. Our findings demonstrated that *AR* variants exert heterogeneous effects on receptor function, with variable impairments in protein expression, nuclear localization, and transcriptional activity. Notably, nuclear localization showed a significant positive correlation with transcriptional activity, suggesting that impaired nuclear trafficking represents an important mechanism contributing to AR dysfunction. Furthermore, residual AR transcriptional activity was positively correlated with EMS scores, indicating that remaining receptor activity contributes to the clinical severity of AIS. These findings highlight the importance of integrating functional characterization with clinical phenotyping to improve the interpretation of *AR* variants and better understand the molecular basis underlying phenotypic variability in AIS.

To date, more than 800 *AR* variants distributed across different protein domains have been reported. Affected individuals may present with normal phenotypes or varying degrees of DSD severity. In our cohort, patients with CAIS generally showed a more consistent clinical phenotype, whereas PAIS patients exhibited greater phenotypic heterogeneity. Although individual *AR* variants did not show a simple one‐to‐one correspondence with clinical severity, variant class appeared to influence the clinical phenotype. Specifically, truncating variants were more frequently associated with CAIS, whereas nontruncating variants were enriched among PAIS patients.

Among the 33 AIS patients carrying *AR* variants, 13 harbored truncating variants. These premature termination codons are presumed to trigger nonsense‐mediated mRNA decay (NMD), a quality control mechanism that selectively degrades transcripts containing premature stop codons, thereby preventing the production of aberrant C‐terminally truncated proteins [12]. Because the molecular mechanisms governing NMD efficiency and specificity remain incompletely understood, and appropriate in vitro systems that faithfully recapitulate key NMD steps are lacking [13, 14], functional validation of truncating variants was not performed in this study. Although NMD generally protects cells from harmful truncated proteins, this mechanism is not universally beneficial. In some genetic disorders, partially functional truncated proteins may mitigate disease severity. For example, in Duchenne muscular dystrophy, NMD eliminates dystrophin transcripts harboring nonsense variants, thereby preventing the production of partially functional dystrophin and exacerbating muscle degeneration [15]. Interestingly, in our cohort, patients carrying truncating *AR* variants presented with either PAIS or CAIS phenotypes. Two patients harboring truncating variants terminating within the NTD exhibited PAIS rather than the classical CAIS phenotype. This observation suggests that partial escape from NMD may occur, allowing residual expression of truncated AR proteins in key target tissues and thereby attenuating phenotype severity.

After reviewing studies published by multiple research groups, we consider that currently available in vitro assays used to assess *AR* variant activity remain imperfect. In some studies, functional assays provide strong experimental evidence supporting pathogenicity, whereas in others, inconsistent results have been reported for identical variants [16–18]. These discrepancies may arise from missing key cofactors in experimental models, resulting in false‐negative or false‐positive findings. To explore how missense variants translate into patient phenotypes, we performed in vitro functional analyses. Notably, the p.G821E variant migrated faster than WT, appearing as an 80 kDa band. To clarify its molecular basis, we focused on subcellular localization and transcriptional activity. Both datasets consistently indicated that p.G821E results in reduced AR functional activity, with partial nuclear translocation upon androgen stimulation but markedly impaired transcriptional activation, consistent with the observed PAIS phenotype.

Our study further demonstrated a significant positive correlation between AR transcriptional activity and EMS scores, suggesting that residual AR transcriptional function represents an important molecular determinant contributing to the clinical severity of AIS. In addition, AR transcriptional activity was positively correlated with nuclear localization capacity, further supporting that proper nuclear trafficking of AR is an essential step for maintaining its transcriptional function. However, the functional consequences of individual *AR* variants do not always correspond directly to clinical phenotypes. For example, the p.Q734K variant was identified in both CAIS and PAIS individuals. Although this variant retained relatively high residual transcriptional activity in vitro, one individual carrying this variant presented with a CAIS phenotype. These findings suggest that residual AR function can partially explain phenotypic variability among individuals with AIS but is insufficient to fully predict the clinical presentation of individual patients.

In this cohort, PAIS patients predominantly carried missense variants. Most missense variants showed varying degrees of impairment in nuclear localization or transcriptional activity, further supporting the association between altered AR molecular function and PAIS phenotype. However, several variants, including p.P672L, did not show obvious functional impairment in the current in vitro assays, whereas p.G578E and p.V888A exhibited increased transcriptional activity. These findings indicate that altered transcriptional activity alone may not fully explain the clinical phenotype. Functional evaluation using cellular models may not fully capture the complexity of AR activity within the in vivo physiological context. Additional factors, including regulatory region variants, aberrant splicing events, alterations in *AR* co‐regulators, or other genetic modifiers, may contribute to the modulation of androgen signaling and further influence the clinical manifestations of AIS.

By systematically assessing protein expression, nuclear localization, and transcriptional activity, we delineated the functional consequences of *AR* variants on receptor abundance, subcellular distribution, and downstream transcriptional regulation. These findings highlight multiple mechanistic layers through which *AR* variants disrupt androgen signaling and provide important insights for future mechanism‐based therapeutic strategies. Reduced protein expression suggests impaired folding or stability, rendering variants more susceptible to degradation within the proteostasis network. For such variants with residual function, pharmacological chaperones may improve AR stability and enhance activity [19]. Nuclear translocation defects indicate disrupted dissociation from heat shock proteins, such as HSP90, or impaired recognition of nuclear localization signals [20], suggesting that strategies enhancing AR‐chaperone interactions or nuclear import may be beneficial [21]. Furthermore, variants exhibiting impaired transcriptional activation despite preserved expression or localization may disrupt ARE binding or coactivator recruitment, supporting therapeutic approaches targeting *AR* coactivator networks or downstream gene regulation. Overall, our results emphasize that functional impairment caused by AIS‐associated *AR* variants is multilevel and heterogeneous. Different variants disrupt androgen signaling at distinct functional nodes. This stratified analysis of protein expression, nuclear localization, and transcriptional activity provides a framework for individualized, mechanism‐oriented therapeutic strategies.

## 5. Conclusion

In conclusion, this study demonstrates that AIS‐associated *AR* variants cause diverse molecular functional alterations involving protein expression, nuclear localization, and transcriptional activity. Although variant location within functional domains alone was insufficient to explain clinical variability, truncating variants showed a higher tendency to be associated with CAIS. Importantly, nuclear localization capacity was positively correlated with transcriptional activity, and residual transcriptional activity was associated with EMS scores, highlighting the contribution of remaining AR function to phenotypic severity in AIS. These findings emphasize the importance of integrating genetic, functional, and clinical information for the interpretation of *AR* variants and the individualized assessment of patients with AIS.

## Author Contributions

C.X.G., D.M., and L.J.F. designed the study and were responsible for patient selection. D.M. performed the laboratory work. Y.N.Z. supervised the laboratory work.

## Funding

No funding was received for this manuscript.

## Disclosure

C.X.G. and D.M. have full access to all of the data in the study and take responsibility for the integrity of the data and its accuracy. All authors read, corrected, and made a significant contribution to the manuscript.

## Ethics Statement

This study protocol was reviewed and approved by the Medical Ethics Committee of Beijing Children′s Hospital, Capital Medical University (Approval No. IEC‐C‐008‐A08‐V.05.1 2018‐209). The approved protocol was a retrospective observational study that permitted the review of clinical and genetic data from eligible patients, including those evaluated prior to the date of ethical approval. Written informed consent was obtained from the parents or legal guardians of all participants. The study was conducted in accordance with the Declaration of Helsinki.

## Conflicts of Interest

The authors declare no conflicts of interest.

## Supporting Information

Additional supporting information can be found online in the Supporting Information section.

## Supporting information


**Supporting Information 1** Figure S1: Overview of previously reported missense variants in the androgen receptor. (A) Spatial distribution of *AR* missense variants across the protein domains reported in the literature. (B) Clinical phenotypes and diagnoses of individuals carrying these variants. (C) Summary of functional assay results including androgen binding activity, transcriptional activity, protein expression, and subcellular localization.

**Supporting Information 2** Figure S2: (A) AlphaMissense pathogenicity scores for all missense variants in the AIS cohort. Scores ≥ 0.56 indicate pathogenic/likely pathogenic variants (red), 0.34–0.56 indicate variants of uncertain significance (gray), and ≤ 0.34 indicate benign/likely benign variants (blue). Variants identified in the AIS cohort are indicated. (B) Distribution of the cohort‐identified variants across AR protein domains. (C) Tolerance landscape of AR derived from observed missense and synonymous variants in gnomAD, adjusted for sequence composition. Residue tolerance is color‐coded: red indicates low tolerance (highly intolerant), and blue indicates high tolerance (highly tolerant).

**Supporting Information 3** Figure S3: Characterization of *AR* missense variants identified in the present cohort. (A) Conservation analysis of amino acid residues affected by *AR* missense variants. (B) In silico predictions, clinical phenotypes, and ACMG classification of *AR* missense variants. A total of 23 in silico tools were used to evaluate the potential deleteriousness of each missense variant. The circular plot depicts the distribution of prediction outcomes: yellow, probably significant; red, possibly significant; grey, uncertainly significant. Corresponding clinical phenotypes and ACMG classifications for each variant are displayed alongside the prediction results.

**Supporting Information 4** Figure S4: Quantitative analysis of AR nuclear localization in cells expressing wild‐type or AR variant proteins. Data are presented as mean ± SD. *p* values were determined using a one‐way analysis of variance (ANOVA), and variants were compared with WT. *p<0.05, **p<0.01, ***p<0.001, ****p<0.0001.

**Supporting Information 5** Figure S5: Correlation analysis among AR protein expression, nuclear localization, and transcriptional activity of *AR* variants. Scatter plots showing the relationships between different functional properties of AR variant proteins. (A) Correlation between AR protein expression and nuclear localization. (B) Correlation between AR protein expression and transcriptional activity. (C) Correlation between AR nuclear localization and transcriptional activity. Each dot represents an individual *AR* variant. Correlations were evaluated using Spearman′s rank correlation analysis. The blue line represents the fitted regression line, and the shaded area indicates the 95% confidence interval.

**Supporting Information 6** Description of Table S1: Summary of previously reported functional assays evaluating the pathogenic effects of missense variants. All variants were retrieved from the Human Gene Mutation Database (HGMD Professional). For each variant, the table summarizes the functional assays performed, the cell models used, and the corresponding experimental outcomes.

**Supporting Information 7** Description of Table S2: Summary of in silico predictive scores for the pathogenicity of missense variants. All missense variants identified in the present cohort were systematically assessed using 23 in silico prediction tools to evaluate their potential pathogenicity.

**Supporting Information 8** Description of Table S3: Primers used for construction of *AR* variant expression plasmids. Table S4: Summary of study duration and age range of the research participants. Table S5: Comparison of clinical manifestations between patients with LBD and non‐LBD *AR* variants.

## Data Availability

The data that supports the findings of this study are available in the supporting information of this article.
