# Supplementary material for: Functional Characterization and Genotype‐Phenotype Correlation of Androgen Receptor Variants in Patients With Androgen Insensitivity Syndrome
Source: Hum Mutat. 2026 Oct 5;2026:1882678. doi: 10.1155/humu/1882678 (PMC13636658; doi:10.1155/humu/1882678)
Supplement: Supplementary file 1 [file HUMU-2026-1882678-s003.docx]

**Supplementary Table 3. Primers used for construction of AR variant expression plasmids**

| **Mutation** | **Forward (5** **'-3** **')** | **Reverse (5** **'-3** **')** |
| --- | --- | --- |
| **p. Y269S** | ATGTcCGCCCCACTTTTGGGAGTTCCACCCGC | AAAAGTGGGGCGgACATGCAATCCCCCCGAAG |
| **p. G578E** | CACATGTGaAAGCTGCAAGGTCTTCTTCAAAA | TGCAGCTTtCACATGTGAGAGCTCCATAGTGACA |
| **p. P672L** | GTCAGCtCATCTTTCTGAATGTCCTGGAAGCC | CAGAAAGATGaGCTGACATTCATAGCCTTCAATGT |
| **p. D691E** | GGACACGAgAACAACCAGCCCGACTCCTTTGC | TGGTTGTTcTCGTGTCCAGCACACACTACACC |
| **p. Q734K** | TGGACGACaAGATGGCTGTCATTCAGTACTCCTG | AGCCATCTtGTCGTCCACGTGTAAGTTGCGGA |
| **p. M746K** | TGGGGCTCAaGGTGTTTGCCATGGGCTGGCGA | AAACACCtTGAGCCCCATCCAGGAGTACTGAA |
| **p. Y782N** | GGATGaACAGCCAGTGTGTCCGAATGAGGCAC | ACACTGGCTGTtCATCCGGGACTTGTGCATGC |
| **p. E804V** | CCAGGtATTCCTGTGCATGAAAGCACTGCTAC | TGCACAGGAATaCCTGGGGGGTGATTTGGAGC |
| **p. L806M** | CCAGGAATTCaTGTGCATGAAAGCACTGCTACTC | TGCACAtGAATTCCTGGGGGGTGATTTGGAGC |
| **p. L811V** | TGAAAGCAgTGCTACTCTTCAGCATTATTCCAGTG | GAGTAGCAcTGCTTTCATGCACAGGAATTCCT |
| **p. G821E** | CAGTGGATGaGCTGAAAAATCAAAAATTCTTTGATG | TTTCAGCtCATCCACTGGAATAATGCTGAAGA |
| **p. N834Y** | GtACTACATCAAGGAACTCGATCGTATCATTG | GTTCCTTGATGTAGTaCATTCGAAGTTCATCAAAGAATTTTT |
| **p. Y835H** | GAACcACATCAAGGAACTCGATCGTATCATTG | GTTCCTTGATGTgGTTCATTCGAAGTTCATCAAAGAATT |
| **p. L860F** | TCTACCAGtTCACCAAGCTCCTGGACTCCGTG | CTTGGTGAaCTGGTAGAAGCGTCTTGAGCAGG |
| **p. S885L** | AGTtACACATGGTGAGCGTGGACTTTCCGGAA | GCTCACCATGTGTaACTTGATTAGCAGGTCAAAAGTGAAC |
| **p. V888A** | AAGTCACACATGGcGAGCGTGGACTTTCCGGAA | CTCgCCATGTGTGACTTGATTAGCAGGTCAAA |
| **p. I907L** | AAGTGCCCcAGATCCTTTCTGGGAAAGTCAAGC | AAGGATCTgGGGCACTTGCACAGAGATGATCT |

**Supplementary Table 4. Summary of study duration and age range of the research participants**

| **Parameters** |  |
| --- | --- |
| **Initial visit year (range)** | 2010-2022 |
| **Age at first visit (years), median (range)** | 1.9 (0.3-16.2) |
| **Age at last visit (years), median (range)** | 6.1 (1.3-16.2) |
| **Follow-up duration (years), median (range)** | 1.4 (0-8.8) |

**Supplementary Table 5. Comparison of clinical manifestations between patients with LBD and non-LBD *AR* variants**

| **Clinical manifestation** | **LBD** | **Non-LBD** | **Total** | ***P*-value** |
| --- | --- | --- | --- | --- |
| **Female** | 13 | 5 | 18 | 1.000 |
| **Male** | 10 | 4 | 14 |  |
| **CAIS** | 10 | 5 | 15 | 0.699 |
| **PAIS** | 13 | 4 | 17 |  |
| **Bifid scrotum** | 18 | 6 | 24 | 0.654 |
| **Scrotal fusion** | 5 | 3 | 8 |  |
| **Cryptorchidism** | 14 | 6 | 20 | 0.704 |
| **No cryptorchidism** | 9 | 3 | 12 |  |
| **Distal urethral meatus** | 19 | 7 | 26 | 1.000 |
| **Non-distal urethral meatus** | 4 | 2 | 6 |  |

Note: Patient 15 was excluded from the analysis of external genital manifestations because he was referred after surgical repair of scrotal and urethral abnormalities, which precluded accurate assessment of the original genital phenotype.
